# Ultra-processed food consumption and obesity indicators in individuals with and without type 1 diabetes mellitus: a longitudinal analysis of the prospective Coronary Artery Calcification in Type 1 Diabetes (CACTI) cohort study

**DOI:** 10.1017/S1368980023000848

**Published:** 2023-08

**Authors:** Tiantian Pang, Heewon L Gray, Amy C Alman, Acadia W Buro, Arpita Basu, Shi Lu, Janet K Snell-Bergeon

**Affiliations:** 1 College of Public Health, University of South Florida, Tampa, FL 33612, USA; 2 Moffitt Cancer Center, Tampa, FL, USA; 3 Department of Kinesiology and Nutrition Sciences, University of Nevada at Las Vegas, Las Vegas, NV, USA; 4 College of Public Health and Human Sciences, Oregon State University, Corvallis, OR, USA; 5 Barbara Davis Center for Diabetes, University of Colorado Denver, Aurora, CO, USA

**Keywords:** Ultra-processed food, overweight, obesity, weight gain, waist circumference, type 1 diabetes, cohort studies

## Abstract

**Objective:**

To evaluate the associations of ultra-processed food (UPF) consumption and obesity indicators among individuals with and without type 1 diabetes mellitus (T1DM) from the Coronary Artery Calcification in Type 1 Diabetes cohort study.

**Design:**

A secondary analysis. The consumption of UPF was assessed using the dietary data collected with the Harvard FFQ, and each food item was categorised according to the NOVA food processing classification. Height, weight and waist circumference were measured at baseline and after a mean of 14·6-year follow-up. Generalised estimating equations stratified by diabetes status were used to assess the associations between UPF intake and obesity indicators over 14 years of follow-up.

**Setting:**

USA.

**Participants:**

A total of 600 adults (256 T1DM and 344 non-diabetic controls) aged 39 ± 9·1 years at baseline and followed up for over 14 years were included.

**Results:**

Participants with T1DM consumed significantly more UPF than non-diabetic controls at baseline: 7·6 ± 3·8 *v*. 6·6 ± 3·4 servings per day of UPF, respectively (*P* < 0·01). Participants with T1DM and with the highest UPF intake had the highest weight (*β*_Q4 *v*. Q1_ = 3·07) and BMI (*β*_Q4 *v*. Q1_ = 1·02, all *P* < 0·05) compared with those with the lowest UPF intake. Similar positive associations were observed in non-diabetic controls.

**Conclusions:**

Individuals with T1DM may consume more UPF than non-diabetic controls. Positive associations between UPF consumption and obesity indicators suggest that limiting UPF can be recommended for obesity prevention and management. Further research is needed to confirm these findings.

Overweight and obesity are defined as excessive body fatness that may impair health^(1)^. Obesity affected more than 650 million adults worldwide in 2022^(2)^. In the USA, obesity continues to be a growing epidemic health problem. The prevalence of obesity in the USA has increased markedly since the 1990s, with 41·9 % of US adults being obese in 2017–2020^(3)^. Weight, BMI and waist circumference (WC) are common obesity indicators. Obesity is a complex and multifactorial disease that can be developed when energy intake is continuously higher than energy expenditure, resulting in energy imbalance^(4)^. Dietary intake is among the many risk factors contributing to energy imbalance and eventually leading to overweight or obesity. Obesity is associated with increased mortality, type 2 diabetes mellitus, hypertension, gallbladder disease and coronary heart diseases^(5,6)^. The prevalence of overweight and obesity has also increased dramatically over the past years among people with type 1 diabetes mellitus (T1DM), and T1DM has almost doubled the risk of CVD than the general population^(7,8)^. Thus, investigating modifiable risk factors related to obesity is crucial, especially among individuals with T1DM who are at a higher risk of developing CVD.

The increasing prevalence of obesity is accompanied by an increased intake of energy-dense foods that are high in fat and sugar^(9)^. In addition, emerging evidence indicates that food processing rather than individual nutrients may be independently associated with the risk of obesity^(10)^. A categorisation of food processing, NOVA (not an acronym), has been established by Monteiro et al. and utilised by several studies^(11)^. Ultra-processed foods (UPF) are characterised as industrial formulations mostly from substances derived from foods or synthesised ingredients and made with no or minimal whole foods by the NOVA classification system^(11)^. Consumption of UPF contributed to over half of the total daily energy intake in the USA between 2007 and 2012^(12)^. In contrast to whole foods or minimally processed foods, UPF are hyper-palatable, ready-to-eat, shelf-stable and energy-dense^(11,13)^. A recent randomised controlled trial suggested that UPF consumption increased energy intake and led to weight gain^(10)^. Diets high in UPF have also been linked with an increased risk of all-cause mortality, coronary heart diseases, metabolic syndrome and overall cancer^(14)^. Thus, UPF intake can be a potent marker for assessing diet quality and associated health outcomes.

Several cross-sectional and prospective studies have investigated the associations between UPF intake characterised by NOVA and overweight and obesity^(15–23)^. However, most studies used data from the general population, with little or no attention given to people with T1DM^(15–18)^. To date, only one cohort study used data collected from pregnant women with pre-existing diabetes mellitus (45·2 % had T1DM and 47·6 % had type 2 diabetes mellitus), and they found that UPF consumption increased gestational weight gain^(21)^. Given that UPF dominate the energy intake in the American diet and individuals with diabetes are at a higher risk of developing obesity- and diabetes-related complications, the importance of studying the impact of UPFs on obesity among these high-risk populations is paramount. To the best of our knowledge, no study has yet investigated these prospective relationships in participants with and without T1DM. Thus, we aimed to explore the associations between UPF consumption characterised by NOVA and obesity indicators in individuals with and without T1DM that were followed up for over 14 years from the Coronary Artery Calcification in Type 1 Diabetes (CACTI) study^(24)^. We hypothesised that higher UPF intake would be positively associated with obesity indicators in this population.

## Methods

### Study population

The CACTI study is a prospective cohort study that is designed to examine coronary artery calcification among adults with and without T1DM. Details of the study design and participant characteristics have been published previously^(24)^. In the original study, 1416 participants (652 with T1DM and 764 non-diabetic controls) were recruited at baseline between March 2000 and April 2002, and the cohort was re-examined 14·6 (12–18) years after baseline assessment. Participants with T1DM were insulin dependent within 1 year of diagnosis, had a clinical course consistent with T1DM and had a diabetes duration of more than 4 years. Non-diabetic controls had fasting blood glucose < 110 mg/dl and had no history of diabetes diagnosis. The present study conducted a secondary analysis using data collected from the baseline and year 14 follow-up of the CACTI study. A total of 248 participants were excluded due to missing weight or WC data and incomplete FFQ at baseline. We also excluded 568 participants who did not attend or were missing weight or WC data at the 14-year follow-up visit. The final sample consists of 600 participants, including 256 T1DM and 344 non-diabetic controls.

### Dietary assessment

Food consumption was evaluated at baseline and 14-year follow-up through a previously validated 131-item Harvard semi-quantitative FFQ^(25)^. Dietary data collected at both visits were included in the analyses. Two versions of the Harvard FFQ were used, 1988 Harvard FFQ for baseline visit and Grid 2007 FFQ for the 14-year follow-up visit. The questions are similar in these two FFQ, and we included the common food items from both questionnaires for consistency analyses. The FFQ collected the frequency of food consumption for each food item during the past 12 months (with 9 frequency options: ‘never or less than once per month’, ‘1–3 times per month’, ‘1 per week’, ‘2–4 per week’, ‘5–6 per week’, ‘1 per day’, ‘2–3 per day’, ‘4–5 per day’ and ‘6+ per day’). Standard portion sizes were provided in natural units (e.g. slice of bread, one apple, one egg, one can of soda) or in household measures (e.g. cup, ounce, teaspoon)^(25)^. Daily food consumption was calculated by multiplying the frequency of food consumption by the standard portion sizes. For example, a response of ‘5–6 per week’ consumption of one orange was estimated as 0·8 servings per day. Based on many previously published studies, servings per day are a more appropriate unit for the estimation of dietary intake using data collected from FFQ^(19,26)^. Each food item was classified according to the NOVA classification system into unprocessed or minimally processed foods (Group 1); processed culinary ingredients (Group 2); processed foods (Group 3) or UPF (Group 4) (online Supplementary Table)^(11)^. For the purpose of this study, only food items from Group 4 were included. The frequency of UPF consumption was estimated with the use of the sum of food items from Group 4 in the FFQ (total of forty-three items). UPF were categorised into quartiles according to total consumption (servings per day).

### Outcome assessment

Physical exam measurements included height, weight and WC measured at baseline and 14-year follow-up. Weight was measured on a calibrated detector scale to the nearest 0·1 kg twice and averaged. Study participants were asked to wear an examination gown or minimal clothing to ensure accurate weight measurement. Height was measured using a calibrated stadiometer to the nearest 0·1 cm twice and averaged. WC was measured over bare skin or an examination gown at the smallest point between the iliac crest and the 10th rib to the nearest 0·1 cm twice and averaged. BMI was calculated in kg/m^2^. Obesity was defined as BMI ≥ 30 kg/m^2^, and overweight including obesity was defined as BMI ≥ 25 kg/m^2^ by the WHO^(2)^.

### Statistical analyses

Baseline characteristics of study participants were described as mean ± sd for continuous variables and frequencies (percentages) for categorical variables, according to T1DM status and UPF quartiles. The main sources of UPF presented as mean ± sd were compared at baseline and 14-year follow-up by T1DM status. Generalised estimating equations with repeated measures were used to assess the associations of UPF quartiles (lowest quartile was the reference group) with the following obesity indicators: weight (kg), WC (cm) and BMI (kg/m^2^) were continuous, and obesity and overweight including obesity were binary. Generalised estimating equations modeling is a robust method that takes into consideration the longitudinal nature of the study with repeated measurements of both outcomes and exposures and correlated observations. Estimated regression coefficients with standard errors for continuous variables and log-odds with standard errors for binary variables were presented for generalised estimating equations models. Covariates, including general demographic factors such as age, sex, race and education and behavioural factors such as physical activity and smoking, were included in the analyses. We also adjusted for diabetes duration, antihypertensive and lipid-lowering drugs since they might affect obesity outcomes^(27)^. Models were adjusted for potential covariates hierarchically as follows: model 1 was unadjusted; model 2 was model 1 adjusted for sex, age, race, education, smoking, physical activity and duration of follow-up; model 3 is model 2 plus total energy intake and model 4 is model 3 plus antihypertensive and lipid-lowering drugs and diabetes duration for T1DM. Stratified analyses by diabetes status were conducted since the research interest is to assess the associations between UPF and obesity among T1DM. All analyses were performed using SAS 9·4 (SAS Institute Inc.). Significance was defined at a two-sided *P* < 0·05.

## Results

A total of 600 participants (256 with T1DM and 344 non-diabetic controls) were included in the analyses. The mean age of participants was 39 ± 9·1 (mean **±** sd) years at baseline and the mean follow-up time was 14·6 ± 1 years. Among participants with T1DM, the prevalence of overweight including obesity has increased from 52 % at baseline to 61 % at 14-year follow-up, and obesity prevalence has increased from 15 % to 24 %. Similar increasing trends and percentages of overweight and obesity were observed among non-diabetic controls (overweight including obesity increased from 53 % to 59 %; obesity increased 16 % from to 23 %). Baseline characteristics of participants according to T1DM status and UPF quartiles are presented in Table 1. Participants with T1DM were more likely to be younger White females, had better lipid profiles and lower diastolic blood pressure, were on antihypertensive and lipid-lowering drugs and consumed more UPF (servings per day) than those without diabetes. Participants in the highest UPF quartile were more likely to be White males with T1DM, were on antihypertensive and lipid-lowering drugs, were overweight and had higher total energy intake, BMI, weight, WC, fasting glucose and blood pressure than those in the lowest quartile.

**Table 1:** Baseline characteristics of participants according to diabetes status and UPF quartiles (*n* 600)^*^

	T1DM (*n* 256)		Non-diabetic control (*n* 344)			Q1 (*n* 150)		Q2 (*n* 150)		Q3 (*n* 150)		Q4 (*n* 150)		
Characteristics	Mean	sd or %	Mean	sd or %	*P*	Mean	sd or %	Mean	sd or %	Mean	sd or %	Mean	sd or %	*P*
Age (years)	36·5	9·2	41	8·5	< 0·01	39·2	9	39	9·6	39·6	8·8	38·5	8·4	0·78
Sex (male)	95	37	171	50	0·01	52	35	54	36	69	46	91	61	< 0·01
Race (White)	252	99	291	85	< 0·01	126	84	136	91	139	93	142	95	0·01
Education (years)	17·1	15	16·2	2·4	0·28	15·7	8·4	16·2	2·2	16·1	2·3	18·2	7·2	0·14
Smoking status					0·77									0·66
Current	17	7	19	6		9	6	9	6	9	6	9	6	
Past	53	20	77	23		32	21	41	27	29	19	28	19	
Never	186	73	246	71		108	72	100	67	111	75	113	75	
Physical activity	67	26	78	23	0·32	31	21	37	25	35	23	42	28	0·52
T1DM	N/A	N/A	N/A	N/A		51	34	61	41	60	40	84	56	0·01
T1DM duration (years)^†^	22·6	8·6	N/A	N/A		25·0	9·97	21·9	8·4	21·5	8·6	22·5	7·8	0·06
Energy intake (kcal/d) (1 kcal = 4.184 kJ)	2017	827	1965	706	0·41	1696	693	1949	558	1948	713	2355	912	< 0·01
BMI (kg/m^2^)	25·9	4·3	25·8	4·4	0·88	25·3	3·9	25·3	4·2	25·9	4·9	26·9	4·1	< 0·01
Weight (kg)	75·3	15·6	77·2	16·9	0·16	72·5	14·7	73·7	14·8	76·8	17·3	82·7	16·7	< 0·01
Waist circumference (cm)	83·8	12·4	85·7	13·6	0·09	81·9	12·2	82·7	11·6	85·1	14·1	90	13·1	< 0·01
Obesity	39	15	56	16	0·73	18	12	17	11	29	19	31	21	0·05
Overweight	134	52	183	53	0·84	75	50	69	46	72	48	101	67	0·01
HbA1c (%)	7·9	1·2	5·5	0·4	< 0·01	6·2	1·3	6·3	1·3	6·5	1·5	7	1·7	< 0·01
Antihypertensive drug	87	34	22	6	< 0·01	22	15	19	13	29	19	39	26	0·02
Lipid-lowering drug	39	15	18	5	< 0·01	6	4	8	5	14	9	29	19	0·02
Unprocessed food consumption (servings/d)	9·0	3·8	9·0	3·7	0·9	9·0	4·2	8·3	3·6	9·2	3·9	9·4	3·4	0·11
Total UPF consumption (servings/d)	7·59	3·83	6·5	3·43	< 0·01	3·3	0·74	5·2	0·5	7·4	0·8	12·2	2·7	< 0·01

UPF: ultra-processed food; Q: quartiles; T1DM: type 1 diabetes mellitus.

* *P* was derived from Student’s *t* test or ANOVA test for continuous variables and χ^2^ test for categorical variables.

^†^For T1DM only.

The main sources of UPF are shown in Table 2. Participants with T1DM had significantly higher UPF intake than non-diabetic controls at baseline (7·59 ± 3·83 (mean ± sd) servings/d in T1DM and 6·55 ± 3·43 servings/d in non-diabetic controls) and 14-year follow-up (5·58 ± 3·37 servings/d in T1DM and 4·63 ± 2·74 servings/d in non-diabetic controls). Soft drinks (including both regular and low-calorie soft drinks), savoury snacks (i.e. chips, French fries, crackers and popcorn) and margarine were consumed more among T1DM than the controls at both time points. In addition, people with T1DM consumed less baked goods at baseline but more processed meat at the follow-up visit than non-diabetic controls.

**Table 2:** Main sources of UPF in participants at baseline and 14-year follow-up by diabetes status^*^

	Baseline					14-year follow-up				
	T1DM (*n* 256)		Non-diabetic controls (*n* 344)			T1DM (*n* 256)		Non-diabetic controls (*n* 344)		
Foods (servings/d)	Mean	sd	Mean	sd	*P*	Mean	sd	Mean	sd	*P*
Processed meat^†^	0·40	0·44	0·34	0·38	0·07	0·26	0·30	0·20	0·21	< 0·01
Soft drinks^‡^	1·48	1·61	0·85	0·97	< 0·01	1·24	1·63	0·61	0·86	< 0·01
Breakfast cereals	0·40	0·52	0·41	0·64	0·4	0·22	0·43	0·19	0·28	0·2
Breads	1·01	0·99	0·90	0·97	0·14	0·67	0·70	0·59	0·61	0·18
Sweets^§^	0·89	0·78	0·96	0·87	0·35	0·67	0·60	0·71	1·02	0·47
Baked goods^||^	0·88	0·68	1·07	0·96	< 0·01	0·63	0·61	0·79	0·89	0·3
Savoury^¶^	0·97	1·02	0·68	0·70	< 0·01	0·74	0·74	0·58	0·46	< 0·01
Margarine	0·52	0·70	0·38	0·53	0·01	0·21	0·45	0·13	0·28	0·02
Other**	1·04	1·00	0·96	0·98	0·39	0·94	0·99	0·83	0·87	0·2
Total UPF	7·59	3·83	6·55	3·43	< 0·01	5·58	3·37	4·63	2·74	< 0·01

UPF: ultra-processed foods; T1DM: type 1 diabetes mellitus.

* *P* was derived from Student’s *t* test.

^†^Hot dog, sausage, salami, bologna and hamburger.

^‡^Sugar-sweetened beverages, low-calorie soft drinks, sports drinks and fruit drinks.

^§^Chocolate, candy, ice cream, jams, jellies and syrup.

^||^Muffins, brownies, doughnuts, bagels, sweet roll, pie, pancake, waffles, cookies and biscuits.

^¶^Chips, French fries, crackers and popcorn.

** Pizza, peanut butter, mayonnaise or other creamy salad dressing, cream cheese, non-dairy coffee whitener and liquor.

The associations between quartiles of UPF consumption and obesity indicators (weight, WC, BMI, overweight and obesity) by T1DM status in the cohort followed up for over 14 years are presented in Table 3. For T1DM, participants who consumed the highest amount of UPF (the fourth quartile) had a higher risk of increasing weight and BMI in the fully adjusted longitudinal models controlled for sex, age, race, education, smoking status, physical activity, duration of follow-up, total energy intake, diabetes duration and antihypertensive and lipid-lowering drugs (weight (*β* ± se): *β*_Q4 *v.* Q1_ = 3·07 ± 1·27; BMI: *β*_Q4 *v.* Q1_ = 1·02 ± 0·40, all *P* < 0·05). The results suggested that T1DM participants with the highest UPF intake had a 3·07 kg higher weight and 1·02 kg/m^2^ higher BMI than those who consumed the least amount of UPF over the 14 years of follow-up. However, no statistically significant association was observed for WC, overweight or obesity. Among non-diabetic controls, weight, WC, BMI and overweight were higher among those in the top quartile of UPF intake compared with those in the lowest quartile (weight: *β*_Q4 *v.* Q1_ = 3·36 ± 1·27; WC: *β*_Q4 *v.* Q1_ = 3·80 ± 1·19; BMI: *β*_Q4 *v.* Q1_ = 1·15 ± 0·43, overweight: *β*_Q4 *v.* Q1_ = 0·70 ± 0·25; all *P* < 0·05). Thus, an increase of 0·70 was expected in the log-odds of overweight in UPF quartile 4 compared to quartile 1 among non-diabetic controls. In secondary analyses, we also adjusted for unprocessed food consumption (i.e. mainly fruits and vegetables) in the multivariate models and the associations were not altered, which indicates that the increase in obesity indicators was mainly driven by UPF consumption.

**Table 3:** Associations between quartiles of UPF consumption (in servings/d) and obesity indicators by diabetes status in the CACTI cohort followed up for over 14 years^*^

	T1DM						Non-diabetic controls					
	Q2		Q3		Q4		Q2		Q3		Q4	
	*β*	se	*β*	se	*β*	se	*β*	se	*β*	se	*β*	se
Weight (kg)												
Model 1	0·61	0·97	1·44	1·20	**4·19**	1·23	0·23	0·81	**2·32**	0·89	**3·74**	1·22
Model 2	0·64	0·97	1·46	1·22	**3·08**	1·20	−0·17	0·75	1·32	0·82	**3·13**	1·21
Model 3	0·65	0·97	1·47	1·22	**3·11**	1·21	−0·18	0·74	1·30	0·82	**3·08**	1·27
Model 4	0·36	1·01	1·21	1·27	**3·07**	1·27	−0·08	0·76	1·13	0·80	**3·36**	1·27
WC (cm)												
Model 1	1·42	1·25	2·63	1·44	**4·15**	1·45	2·04	1·08	**2·64**	1·15	**5·04**	1·20
Model 2	2·06	1·13	2·38	1·37	**3·37**	1·40	1·30	0·93	1·61	1·09	**3·30**	1·14
Model 3	2·13	1·13	2·48	1·37	**3·56**	1·43	1·39	0·93	1·33	1·10	**3·68**	1·21
Model 4	2·18	1·23	2·04	1·49	2·89	1·59	1·46	0·93	1·12	1·07	**3·80**	1·19
BMI (kg/m^2^)												
Model 1	0·35	0·34	0·43	0·40	**1·07**	0·42	0·11	0·28	**0·77**	0·33	**1·17**	0·41
Model 2	0·43	0·32	0·71	0·40	**0·97**	0·38	0·02	0·26	0·59	0·30	**1·04**	0·41
Model 3	0·45	0·32	0·73	0·40	**1·02**	0·39	0·03	0·26	**0·60**	0·30	**1·07**	0·43
Model 4	0·42	0·33	0·71	0·40	**1·02**	0·40	0·07	0·26	0·54	0·30	**1·15**	0·43
Overweight												
Model 1	0·07	0·25	−0·16	0·23	0·11	0·23	0·06	0·18	0·17	0·16	**0·65**	0·21
Model 2	0·13	0·25	−0·17	0·25	0·01	0·24	0·12	0·19	0·18	0·19	**0·62**	0·23
Model 3	0·17	0·27	−0·13	0·27	0·02	0·26	0·13	0·19	0·21	0·19	**0·69**	0·24
Model 4	0·19	0·27	−0·14	0·27	0·05	0·27	0·12	0·19	0·22	0·19	**0·70**	0·25
Obesity												
Model 1	0·14	0·33	**0·64**	0·31	0·44	0·28	0·04	0·22	0·29	0·21	0·27	0·26
Model 2	0·25	0·36	**0·71**	0·33	0·40	0·31	−0·03	0·24	0·27	0·22	0·25	0·26
Model 3	0·28	0·36	**0·75**	0·34	0·49	0·35	−0·04	0·24	0·24	0·22	0·18	0·27
Model 4	0·32	0·36	0·66	0·35	0·34	0·36	0·01	0·25	0·27	0·23	0·21	0·28

UPF: ultra-processed food; Q: quartiles; T1DM: type 1 diabetes mellitus; WC: waist circumferences.

* Generalised estimating equations were used to measure parameter estimates and se of obesity indicators. Q1 is reference category. Model 1 was unadjusted model. Model 2 adjusted for sex, age, race, education, smoking status, physical activity, and duration of follow-up. Model 3 is model 2 further adjusted for total energy intake. Model 4 is model 3 further adjusted for diabetes duration, antihypertensive and lipid-lowering drugs for T1DM; antihypertensive and lipid-lowering drugs for non-diabetic control.

Significant difference at *P* < 0·05 (bold).

## Discussion

In this longitudinal analysis using data collected from 600 participants followed up for over 14 years in the CACTI study, higher UPF consumption was positively associated with weight and BMI among individuals with T1DM and with weight, WC, BMI and overweight among non-diabetic controls, and these findings were independent of total energy intake. Moreover, individuals with T1DM consumed more UPF than those without diabetes at baseline and 14-year follow-up. Overall, our study revealed that T1DM consumed more UPF over time, and increased UPF consumption was positively associated with obesity indicators among participants with and without T1DM.

As far as we know, this is the first study that investigated and suggested a positive link existed between UPF intake characterised by NOVA and obesity indicators among individuals with T1DM. Overweight and obesity are becoming more common in individuals with T1DM^(28)^. Our cohort reinforced this rising trend as the prevalence of obesity in T1DM has increased from 15 % at baseline to 24 % at year 14, and the prevalence of overweight including obesity has increased from 52 % to 61 %. Obesity in people with T1DM contributes to an elevated risk of both diabetes-related and obesity-related complications, such as CVD, heart failure, various cancers and mortality^(29)^. Given the large UPF consumption in the USA (almost 60 % of caloric intake) and the crucial role of diet in diabetes management, our findings suggested that T1DM-specific recommendations should be developed to reduce UPF intake and prevention and management of obesity and its related complications^(12)^.

Our results suggested positive associations between UPF and obesity indicators, which were consistent with previous prospective cohort studies^(30–32)^. A recent randomized controlled trial suggested that participants gained 0·9 ± 0·3 kg weight (*P* = 0·009) after 2 weeks of UPF diets^(10)^. Another multi-national cohort study also found a 1 sd increment of UPF consumption was associated with a weight gain of 0·12 kg per 5 years^(32)^. Results from a French cohort showed that UPF were associated with BMI gain in participants^(30)^, and another Brazilian prospective study found UPF consumption was associated with weight gain^(31)^. Although we did not find significant associations between UPF and risk of overweight and obesity in T1DM, consistent weight gain caused by a long-term UPF consumption may increase the risk of overweight or obesity. In addition, we observed that higher UPF consumption was associated with higher systolic and diastolic blood pressure, suggesting that UPF may also have a negative impact on cardiovascular health.

UPF tend to be nutrient-poor but energy-dense and high in fats, Na, sugar, preservatives and additives; hyper-palatable; ready-to-eat and have a long shelf life, according to NOVA classification^(33)^. Individuals who consume more UPF may have increased energy intake and disrupted satiety signaling than those with a minimally processed diet^(10)^. UPF are also designed to have enhanced flavor and pleasant texture and people tend to consume more in a shorter time, resulting in an increased eating rate, excessive consumption and delayed satiety^(34)^. In addition, UPF often contain emulsifiers and artificial ingredients, which may have pro-inflammatory effects that are associated with chronic diseases such as obesity^(35,36)^. All these features of UPF could explain their positive associations with obesity indicators.

In our study, participants with T1DM consumed 7·59 servings per day of UPF at baseline and 5·58 at 14-year follow-up, which was significantly higher than the amount of UPF intake in non-diabetic controls. Although it is believed that T1DM should have a healthier diet, previous studies have found that T1DM consumed more saturated fat, had higher pro-inflammatory diets and had insufficient micronutrient intake^(27,37,38)^. Among the main sources of UPF, soft drinks, savoury snacks, margarine and processed meat were significantly higher in T1DM than the controls. Soft drinks are loaded with sugar, and their positive associations with weight gain have been well-established^(39)^. Low-calorie beverages (i.e. diet coke) are a type of soft drink that contains artificial sweeteners. Numerous cohort studies have shown that consumption of artificial sweeteners was positively associated with BMI, WC and obesity, even though they do not contain any sugar or calories^(40)^. Studies also suggested that artificial sweeteners have been associated with gut microbiome dysbiosis and CVD^(35,36)^, indicating that food processing rather than individual nutrients may affect health outcomes. Savoury snacks, including popcorn and French fries, are high in saturated fats and Na, which may exert obesogenic effects^(41)^. Margarine is a highly processed food mainly made from vegetable oil, but it often goes through a hydrogenation process to harden its vegetable oil contents, and unhealthy trans-fat might be produced as a by-product^(42)^. Trans-fat intake has been linked with weight gain and obesity, potentially through the development of insulin resistance^(43,44)^. In addition to trans-fat, margarine may also contain emulsifiers and other food additives, and these ingredients may contribute to weight gain^(45)^.

The study has some limitations. First, dietary data were collected from a retrospective self-report FFQ and recall bias may exist. Second, the FFQ was not designed to classify foods based on the NOVA classification, and misclassification may be present in our methodology. However, two authors (authors 1 and 4) independently coded the food items using NOVA categories and agreed on the final classification. Third, our findings were only generalisable to US adults with T1DM and those without diabetes. Fourth, the sample size is relatively small at year 14 compared to the baseline. The loss to follow-up may drive the results toward null findings. However, further analyses showed that the baseline demographic characteristics of those participants who lost to follow-up were similar and not significantly different from those who remained in the study. Last, obesity indicators including weight and BMI may not be sufficient to assess body fatness. Skinfold tests or DXA scans are often required to examine body composition accurately and diagnose overweight or obesity. This study also has some strengths. To our knowledge, this is the first longitudinal analysis that evaluated the associations between UPF consumption and obesity indicators in individuals with and without T1DM. Furthermore, we incorporated diet data collected at both time points (baseline and 14-year follow-up) into our analyses. Moreover, the anthropometric measurements were performed by trained staff in a clinical setting.

Although the observed relationships need to be further investigated in ethnically diverse populations (i.e. underserved population and minority with potentially higher UPF intake) and other study settings, our study has potential implications for obesity prevention and could contribute to future studies. Our study suggests an urgent need for implementing population-wide strategies such as encouraging the consumption of unprocessed/minimally processed foods and requiring warning labels on UPF packaging.

In conclusion, our longitudinal analysis of the prospective CACTI cohort study suggests that increased UPF consumption was positively associated with obesity indicators among participants with and without T1DM. Policies and recommendations aiming at reducing the consumption of UPF may help prevent obesity, especially for T1DM who are at a higher risk of developing both diabetes-related and obesity-related complications.

Data sharing: Data described in the manuscript, codebook and analytic code will not be made available because our participants only provided their informed consent for the use of their data by the original research team.
